# Non-Destructive Mangosteen Volume Estimation via Multi-View Instance Segmentation and Hybrid Geometric Modeling

**DOI:** 10.3390/jimaging12010001

**Published:** 2025-12-19

**Authors:** Wattanapong Kurdthongmee, Arsanchai Sukkuea, Md Eshrat E Alahi, Qi Zeng

**Affiliations:** 1 School of Engineering and Technology, Walailak University, Nakhon Si Thammarat 80160, Thailand; 2 Shenzhen Institutes of Advanced Technology, Chinese Academy of Sciences, Shenzhen 518055, China; 3 College of Physics and Optoelectronic Engineering, Shenzhen University, Shenzhen 518061, China

**Keywords:** mangosteen, volume estimation, instance segmentation, computer vision, precision agriculture, multi-view imaging, geometric modeling, machine learning

## Abstract

In precision agriculture, accurate, non-destructive estimation of fruit volume is crucial for quality grading, yield prediction, and post-harvest management. While vision-based methods provided some usefulness, fruits with complex geometry—such as mangosteen (*Garcinia mangostana* L.)—are difficult due to their large calyx, which may lead to difficulties in solving using traditional form-modeling methods. Traditional geometric solutions such as ellipsoid approximations, diameter–height estimation, and shape-from-silhouette reconstruction often fail because the irregular calyx generates asymmetric protrusions that violate their basic form assumptions. We offer a novel study framework employing both multi-view instance segmentation and hybrid geometrical feature modeling to quantitatively model mangosteen volume with traditional 2D imaging. A You Only Look Once (YOLO)-based segmentation model was employed to explicitly separate the fruit body from the calyx. Calyx inclusion resulted in dense geometric noise and reduced model performance ($R^{2}<0.40$). We trained eight regression models on a curated and augmented 900 image dataset (N=720, test N=180). The models used single-view and multi-view geometric regressors ($V\propto A^{1.5}$), polynomial hybrid configurations, ellipsoid-based approximations, as well as hybrid feature formulations. Multi-view models consistently outperformed single-view models, and the average predictive accuracy improved from $R^{2}=0.6493$ to $R^{2}=0.7290$. The best model is indeed a hybrid linear regression model with side- and bottom-area features—($A_{s}^{1.5}$, $A_{b}^{1.5}$)—combined with ellipsoid-derived volume estimation—($V_{\mathrm{ellipsoid}}$)—which resulted in $R^{2}=0.7290$, a Mean Absolute Percentage Error (MAPE) of 16.04%, and a Root Mean Square Error (RMSE) of 31.9 ${\mathrm{cm}}^{3}$ on the test set. These results confirm the proposed model as a low-cost, interpretable, and flexible model for real-time fruit volume estimation, ready for incorporation into automated sorting and grading systems integrated in post-harvest processing pipelines.

## 1. Introduction

Mangosteen (*Garcinia mangostana* L.) is a tropical high-value fruit extensively grown in Southeast Asia, identified by its flavor, nutritional profile, and distinctive morphology [1]. Quality evaluation in export markets mainly relies on characteristics like color, texture, size, and volume, which impact the price and consumer acceptance [2]. Fruit volume is still one of the key attributes to measure maturity, internal quality, and edible yield among these properties [3]. Conventional volume estimation methods, such as water displacement and manual calliper measurement, are destructive, labor-intensive, and unsuitable for high-throughput post-harvest processes [4,5]. These considerations have sparked interest in computer vision and machine learning as non-destructive, scalable approaches to fruit quality monitoring [6,7,8,9].

Nonetheless, 2D images are not good for volume estimation in fruits with complex morphology. The quasi-spherical pericarp and ribbed, highly variable calyx of mangosteen generate shape irregularities that violate classical geometric notions such as ellipsoid or cylindrical models [10,11,12]. Single-view imaging also restricts geometric inference in terms of providing information about depth and lateral asymmetries [13]. Multi-view reconstruction and deep learning-based volumetry have shown great promise in agriculture [14,15,16], yet they remain underutilized for composite fruits whose morphological components vary greatly in terms of shape and structural consistency.

Volumetric estimation by means of limited 2D imagery is also an on-going area of research in the field of medicine and biomaterials, where deep regression, multi-view inference, and hybrid geometric-learning were applied to study the morphology of organs, tumor boundaries, and microstructures [17,18,19]. These advancements show the promise of reliable 3D estimation without the need for special hardware, but in an agricultural context, uncontrolled light, fluctuating natural textures, and the requirement of low-cost imaging systems become more significant challenges.

Hence, such considerations have led to a need for a domain-specific solution of mangosteen volumetry using contemporary segmentation and multi-view modeling approaches, yet without sacrificing field and packhouse applicability.

In this paper, we present a new volumetric modeling protocol for mangosteen, where multi-view instance segmentation is combined with hybrid geometric modeling. Its key contributions are as follows:

- A multi-view instance segmentation pipeline for composite fruit morphology. We construct a You Only Look Once (YOLO)-based segmentation model capable of distinguishing the mangosteen body, the calyx, and the calibration marker. It allows explicit quantification of the effects the irregular and lobed geometry of the calyx have on volumetric estimation. To our knowledge, this is the first structural segmentation framework to isolate calyx regions for downstream volumetric estimation.
- Assessment of geometric completeness across the perspective of a single view versus multiple views. In contrast to previous papers, which are limited to lateral views, we compare the presence of side view, bottom view, and combined view to quantify how multi-view shape-modeling cues affect volume estimation. Such a comparison provides general practical implications for the best images of composite tropical fruits under which we model them.
- A hybrid geometry–regression simulation modeling approach for volume estimation. Here, we integrate classical physically motivated models (area-based power laws and ellipsoid approximations) with polynomial regression using multi-view features. This implements a dual-model design that connects classical horticultural geometry to modern predictive modeling and yields a complete analysis of which feature classes produce stable fidelity across fruit batches.
- An effective calibration strategy overcomes distance sensitivity. We propose a per-image scale correction approach, using a calibration marker mounted on every frame. This eliminates fixed-distance calibration and minimizes variations resulting from camera location—facilitating the system to be more workable in true packhouse situations.
- A comprehensive evaluation of when and why calyx geometry makes an impact. By explicitly modeling the calyx region, we describe its influence on volumetric prediction for various models and perspectives. It provides a new empirical perspective of composite fruit morphology and shows why classical ellipsoid-based models cannot predict mangosteen well.

In general, the proposed framework offers an efficient, precise, and domain-specific pipeline for non-destructive mangosteen volume estimation and overcomes some limitations of current agricultural volumetry research.

## 2. Background

Biophysics and fruit volume estimation have evolved from hand-measured fruit volume to machine learning (ML) and multi-view imaging methods. Early papers investigated fruit volumes based on geometric concepts, estimating fruit volumes under geometric assumptions; the estimation was performed with manually determined diameters and heights. Teerachaichayut et al. have employed ellipsoid approximations for mango and mangosteen, and they have found high empirical correlations; however, they had very high manual and controllable parameters [20,21].

But later in our analyses, we also have also found that treating mangosteen as an easy ellipsoid adds enormous geometric asymmetries because of the irregular calyx. The volume is usually overestimated by 8–15% with inclusion of the calyx as part of the boundary, and its width varies by 4–7 mm, depending on calyx size [20,21]. Multi-view approaches showing a similar variation as that of asymmetry caused by the calyx results in 10–18% more reconstruction errors compared to ground truth [13]. It shows that the calyx is not only non-spherical, but also morphologically irregular and must be clearly identified, preventing systematic volumetric error.

The shape-from-silhouette and stereo-vision systems allowed for 3D reconstruction from the space-shift perspective [22,23,24], but they had to deal with issues of lighting change, occlusion, computation resource, and camera calibration [25].

Deep learning has made great strides toward robustness for agricultural vision jobs such as object recognition, segmentation, and fruit phenotyping [26,27,28]. Using YOLO family provides real-time detection ability with high accuracy [29,30,31,32], and more recent segmentation architectures such as the Mask R-CNN, YOLACT, Mask2Former, and SAM provide pixel-wise details of thick fruit boundaries [33,34,35,36]. These advances have allowed more accurate volumetric inference, even in noisy environments [37,38].

Gradually, multi-view imaging and hybrid regression models have been adopted to alleviate the problem of single-view inference [14,15,16]. Regression approaches such as SVR, Random Forests, artificial neural networks, and deep multi-view models have shown very good performance on nonlinear connection between geometrical features and volume [39,40,41,42,43]. However, systematic comparisons of geometric modeling and machine learning methods for composite fruits, especially for fruits with unusual structures such as calyx, are still scarce.

The 2D-to-3D volumetric inference techniques are being applied in medical imaging and biomaterials research, where high accuracy is achieved for organ segmentation, tumor volumetry, and micromechanical reconstruction. Methods including deep regression, self-supervised 3D reconstruction, and generative models outperform even with sparse 2D projections [17,18,19,44,45]. Using these techniques, the results provide methodological insights for better modeling volumetric estimates for agriculture, where full 3D imaging systems are limited.

## 3. Materials and Methods

**Figure 1:**
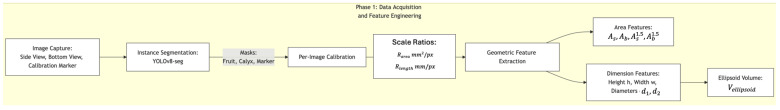
Workflow diagram for Phase 1: image acquisition, instance segmentation, and geometric feature extraction. This phase includes multi-view image capture, YOLOv8-based segmentation, per-image calibration, as well as derivation of area and dimension-based features.

**Figure 2:**
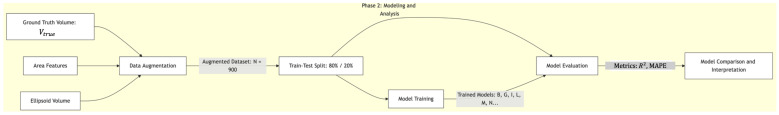
Workflow diagram for Phase 2: data augmentation, regression modeling, and performance evaluation. This phase includes synthetic sample generation, model training and testing, as well as comparative analysis using $R^{2}$ and MAPE metrics.

An organized multi-step pipeline was established to allow for real-world manipulative and non-destructive volume sensing of mangosteen fruit. The overall flow is shown in Figure 1 and Figure 2, which detail the image-based feature extraction approach and the subsequent modeling and evaluation framework. Then, we prepare the fruit samples and ground truth measurement, which is the first step in the pipeline. For finding the physical volume, water displacement is introduced, and density filtering is used to ensure data are correct. The next step would be **multi-view image acquisition**, where each piece of fruit is sampled from two different angles (side and bottom) in a measured lighting. There is also a physical marker for scaling calibration.

Then, an **instance segmentation** model with YOLOv8 is adopted on the fruit body, calyx, and marker. That way, geometrically important areas can be accurately separated from the morphological noise introduced by the calyx, and the relevant features can be separated. The binary masks produced from this operation are input into **geometric feature extraction**, which determines area-based and dimension-based descriptions in physical terms. They include calibrated surface areas, power-law transformations, and ellipsoid volume approximations generated by segmented contours.

Using **data augmentation**, synthetic samples were generated based on the statistical distribution of the cleaned dataset to enhance model robustness and generalizability. This expanded dataset enabled the training of a diverse set of **regression models**, ranging from simple power-law formulations to multi-feature polynomial regressions and ellipsoid-based volume estimators.

Finally, we apply traditional metrics such as the coefficient of determination ($R^{2}$) and Mean Absolute Percentage Error (MAPE) with **performance evaluation** in a test dataset that was not utilized for training. Thus, different feature combinations and regression procedures can be explored, comparing the models in terms of accuracy and reliability.

### 3.1. Fruit Samples and Ground Truth Measurement

We used two sets of data on mangosteen fruit (*Garcinia mangostana* L.). The primary dataset, gathered from a local orchard in Nakhon Si Thammarat, Thailand, in July 2025, comprised 1000 fruits for model building. A distinct validation set, comprising 450 fruits, was utilized for noise robustness testing.

We measured the ground truth volume ($V_{true}$) of the validation set by displacing water [11] and the fresh weight (Mass) with a digital balance. We calculated density ($\rho =\mathrm{Mass}/V_{true}$) and discarded samples with ρ<1.00 g/cm^3^ as measurement errors. After cleaning, 419 valid samples remained in the validation set. Descriptive statistics for the mass and volume of this cleaned dataset are presented in Table 1.

### 3.2. Image Acquisition

Images were acquired with a reproducible laboratory imaging technique. An adjustable rigid stand equipped with a Samsung Slim Fit digital camera (200 mm on top of the imaging plane) is used for mounted vertical cameras. The camera was perpendicular to the platform to reduce perspective distortion and to keep the scale constant amongst samples.

**Figure 3:**
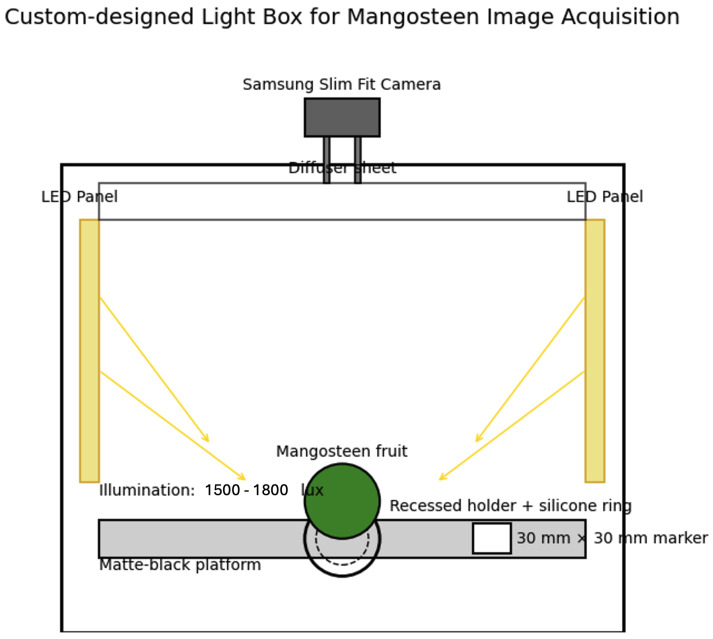
Custom-designed light box used for mangosteen image acquisition. The system includes high-CRI LED panels with diffuser, a fixed top-mounted camera, a matte black platform with a recessed circular holder and silicone ring to stabilize the fruit, as well as a 30 mm × 30 mm calibration marker for scale.

Imaging was carried out in a custom-designed light box (60 cm × 60 cm × 60 cm) containing high-CRI (Color Rendering Index) (Ra > 90) light-emitting panels (LED) mounted on the left and right sides of interior walls. Illumination in the box was 1500 to 1800 lux and was measured onto the platform in a calibrated lux meter. A diffuser sheet had been placed above the LED panels to uniformize the lighting and avoid strong shadows or reflections. The imaging surface was a matte black, non-reflective surface to enhance clarity in the foreground–background at segmentation. Full details of the light box structure, consisting of LED panels, diffuser sheet, camera mount, platform, and calibration marker, are presented in Figure 3.

To obtain complementary geometric information: Each mangosteen fruit was photographed from two orthogonal points of view.

- Side of the Frame: The fruit had the calyx facing the right edge consistently. This shows the height and one lateral dimension of the fruit body.
- Bottom View: On the downside, the bottom view is preferred to the top view, which is heavily dominated by irregular calyx lobes creating occlusions, shadows, and irregular boundaries, as the basal apex of mangosteen is smoother, more circular, and free of protrusions. This provides more stable cross-sectional profile for geometric modeling. The fruit was stabilized via recessed circular holder in (rectified for bottom-view imaging; a diameter of 4.0 cm with a depth of 0.8 cm in the circular holder), which was built onto the platform. The inner rim of the holder was wrapped into a soft silicone ring so as not to roll or have micro-movements, while keeping the calyx clear. It kept the fruit within the center and, in turn, steady during capture in order to ensure the same imaging geometry in all samples.

For each image there were 30 mm × 30 mm physical calibration markers placed in the field of view in order to normalize per-image scaling. This marker was positioned alongside the fruit as they were undergoing scanning, but did not occlude the fruit and was fixed throughout imaging sessions. Photos were obtained with 1920 × 1080 pixels and saved in JPG format.

**Figure 4:**
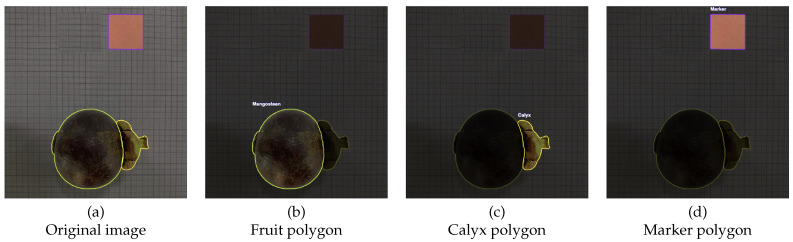
Visualization of side-view image processing stages. (**a**) Original RGB image of mangosteen fruit; (**b**) polygon delineating the fruit region; (**c**) polygon outlining the calyx; (**d**) polygon surrounding the calibration marker (20 mm × 20 mm). The segmentation masks were generated by the YOLOv8-based instance segmentation model trained on annotated mangosteen datasets.

**Figure 5:**
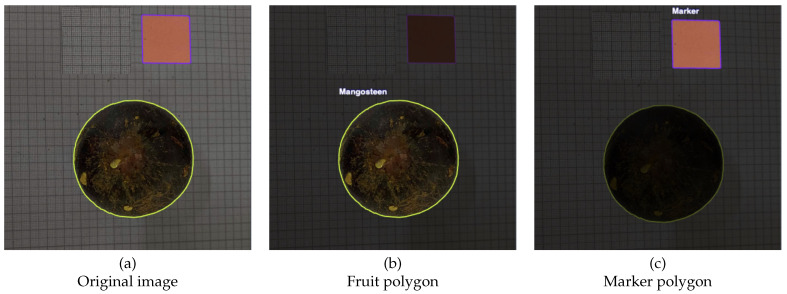
Visualization of bottom-view image processing stages. (**a**) Original RGB image of mangosteen fruit; (**b**) polygon delineating the fruit region; (**c**) polygon outlining the calyx region. These segmentation masks were generated by the YOLOv8-based instance segmentation model trained on the annotated dataset.

Figure 4 and Figure 5 also display sample annotated images from both views. The fruit body, calyx and calibration marker were manually labeled by Roboflow using the Smart Polygon tool [46], which subsequently reset the polygon edges so as to generate more realistic object boundaries on input data. These annotations were afterwards used to train the YOLOv8 instance segmentation model, facilitating the accurate separation of the fruit body, calyx, and marker for subsequent geometric analysis and volume estimates.

### 3.3. Instance Segmentation

YOLOv8 was chosen as the core instance segmentation model since it presents an optimal trade-off between segmentation precision, computational efficiency, and ease of deployment in resource-limited agricultural landscapes. While more heavyweight transformer-based models (e.g., Mask2Former, SAM2) may require large memory, fine-tuning, and/or large-scale prompt-based supervision, YOLOv8 can be trained end-to-end on a modest dataset and run on a standard CPU with real-time performance. SAM2 and associated foundation models can offer good general-purpose segmentation, but they are not designed for small, domain-specific tasks with tightly defined classes (fruit, calyx, marker) and will have to be postprocessed or prompted to obtain stable mask extraction. In contrast, YOLOv8 provides high-quality instance masks directly at minimal overhead, making it more suitable for a practical, scalable pipeline to perform post-harvest mangosteen analysis.

The three vital visual elements in each image considered—the fruit body, calyx, and calibration marker—were automatically separated using an instance segmentation model based on YOLOv8 [47]. The model was constructed using manually annotated mangosteen images in which polygonal masks were drawn for each class. Particular care was taken to remove the calyx from the fruit body mask to reduce geometric noise and improve the reliability of the downstream feature extraction process.

All images were pre-processed using a consistent preprocessing pipeline prior to training. Orientation metadata was automatically corrected and each image was resized to 640 × 640 pixels via stretch interpolation. Histogram equalization was implemented for edge visibility and mask clarity under different illumination.

To provide robustness, extensive data augmentation was employed during training. All original images resulted in three augmented variants, which included several manipulated colour changes (hue, saturation, brightness, and exposure), geometric transformations (horizontal flipping, 90° rotations), and noise (Gaussian blur and salt-and-pepper noise). These augmentation parameters were chosen according to YOLOv8’s best practices and initial validation experiments, which confirmed moderate variations for improving generalization in ways that did not compromise the morphological features essential for accurate segmentation. Table 2 summarises all settings for preprocessing and augmentation.

The side-view and bottom-view images were compared using a confidence threshold of 0.3 for inference. For each target class (‘fruit,’ ‘calyx,’ and ‘marker’), the predicted mask with the largest pixel area was retained, which yields three binary masks per image. Pixels belonging to an object were assigned a value of 255, and background pixels a value of 0. These masks formed the basis of geometric calibration and multi-view feature extraction.

### 3.4. Feature Extraction

Feature extraction processes convert raw image masks into calibrated geometric descriptors, which can be used in regression modeling. Figure 1 provides a view and step-by-step description of the pipeline’s sequence from segmentation output to features derived. Our analysis in Python version 3.10.12 was performed, which is a stable and well-supported platform for scientific image computations. The OpenCV version 4.8.0 library was employed for contour extraction, mask processing, and geometric measurement since this is an optimized and high-performing implementation for computer vision tasks. Pandas was employed for tabular formatting used for organizing, merging, and validating the extracted descriptors to support efficient data manipulation and reproducible analysis. Together, these provide a flexible and computationally effective approach for large-scale, mask-based feature extraction.

1. Pixel Area Calculation: For each binary mask (‘fruit’, ‘calyx’, ‘marker’), the number of non-zero pixels was computed using cv2.countNonZero(). These pixel counts serve as the basis for scale calibration and area estimation.
2. Per-Image Calibration: To convert pixel-based measurements into physical units, a square marker of known area (900 mm^2^) was used. For each image:
  - The area conversion ratio was calculated as $R_{area}=900/A_{marker\_px}$ (mm^2^/px), where $A_{marker\_px}$ is the pixel area of the marker mask.
  - The length conversion ratio was derived as $R_{length}=\sqrt{R_{area}}$ (mm/px), assuming isotropic scaling.
  Images with invalid or zero marker area were excluded from further analysis to ensure calibration integrity.
3. Physical Area Features: The pixel areas of the fruit masks from side and bottom views were converted to physical units:
  - $A_{s}$ and $A_{b}$ represent the fruit surface areas in cm^2^ from the side and bottom views, respectively.
  - Power-law transformations were applied to capture nonlinear relationships: $A_{s}^{1.5}$ and $A_{b}^{1.5}$.
  These features were selected based on prior studies indicating strong correlation between projected area and fruit volume.
4. Dimension Features: To estimate fruit dimensions, the largest contour within each fruit mask was extracted. The cv2.minAreaRect() function was applied to compute the minimum bounding rectangle:
  - The rectangle’s width and height (in pixels) were converted to mm using $R_{length}$ and then to cm.
  - From the side view: height (*h*) and depth estimate 1 ($d_{1}$) were assigned.
  - From the bottom view: width (*w*) and depth estimate 2 ($d_{2}$) were derived using the larger and smaller dimensions, respectively.
  This multi-view approach captures the fruit’s three-dimensional geometry more robustly than single-view estimation.
5. Ellipsoid Volume Approximation: An ellipsoid model was used to approximate fruit volume based on the derived dimensions. As shown in Equation (1), the ellipsoid volume $V_{e}$ is computed using the radii along three orthogonal axes:
  - $r_{h}=h/2$, where *h* is the fruit height from the side view.
  - $r_{w}=w/2$, where *w* is the fruit width from the bottom view.
  - $r_{d}=\left(d_{1}+d_{2}\right)/4$, where $d_{1}$ and $d_{2}$ are depth estimates from side and bottom views, respectively.
  (1) $$V_{e}=\frac{4}{3}\pi r_{h}r_{w}r_{d}$$
  The resulting volume $V_{e}$ is expressed in cm^3^ and serves as a physically interpretable feature for regression modeling.

A summary of all extracted features, their definitions, and units is provided in Table 3. These features ($A_{s}$, $A_{b}$, $A_{s}^{1.5}$, $A_{b}^{1.5}$, *h*, *w*, $d_{1}$, $d_{2}$, $V_{e}$) were compiled into structured datasets for downstream modeling and evaluation.

### 3.5. Data Augmentation

For model robustness, and to prevent overfitting from being worsened, synthetic data samples were set up to give us extra models to handle the processed real dataset. For synthetic sample generation, the original dataset was the statistical baseline by which *N* = 450 valid fruit samples had been selected to generate samples. A subset of core geometric features has been chosen for augmentation involving calibrated area measurements ($A_{s}$, $A_{b}$), dimension estimates (*h*, *w*, $d_{1}$, $d_{2}$), and ellipsoid volume ($V_{e}$).

The means and covariance matrix for these features are calculated from the clean dataset. Using all these parameters, synthetic samples from *N* = 450 were obtained from a multivariate normal distribution using the numpy.random.multivariate_normal() function. This means we maintain the common statistical characteristics among features, by which the variability introduced with the control is managed.

All the generated features were clamped to a minimum threshold of 0.01 in order to make sure that they might be physically plausible so that non-physical zero or negative values could not be extracted. It was then used, as the synthetic sample was concatenated with the original dataset, and the final augmented dataset was constructed by obtaining the N=900 samples. This enlarged dataset was applied to train and test the models for obtaining more stable regression performance through feature fusion.

### 3.6. Regression Modeling

The dataset model (fruits volume ($V_{true}$)) was generated by a vector model. We utilized the scikit-learn library to implement a suite of regression models that evaluated predictability of combinations of different features. The models varied from simple univariate regressions to multivariate and polynomial models, allowing for statistical comparison among contribution to feature definitions.

- Model B (Side-Only Geo): $V\approx k\cdot A_{s}^{1.5}$—a single-feature linear regression based on the power-law transformed side-view area. This model tests the adequacy of $A_{s}^{1.5}$ alone as a volume proxy.
- Model G (Bottom-Only Geo): $V\approx k\cdot A_{b}^{1.5}$—analogous to Model B but based on the bottom-view area. It serves as a baseline for evaluating view-specific predictive strength.
- Model I (Multi-View Geo): $V\approx k_{1}A_{s}^{1.5}+k_{2}A_{b}^{1.5}$—a multivariate linear regression combining both orthogonal views. This model preserves complementary geometric information and explores additive contributions.
- Model L (Poly-Geo): Polynomial regression using $A_{s}^{1.5}$ and $A_{b}^{1.5}$ with degree 2 expansion. We use this model to find out from the area features; we introduce nonlinear interactions in order to strengthen the fit.
- Model M (Ellipsoid): $V\approx k\cdot V_{e}$—a single-feature linear regression model was constructed using ellipsoid volume approximations derived from multi-viewpoint dimensional measurements. This approach yields estimates that are physically interpretable within the context of three-dimensional fruit geometry.
- Model N (Combined Linear): $V\approx k_{1}A_{s}^{1.5}+k_{2}A_{b}^{1.5}+k_{3}V_{e}$—a combined model of area-based and volume-based features. This formulation tests whether $V_{e}$ imparts explanatory power beyond $A_{s}^{1.5}$ and $A_{b}^{1.5}$.

Models O and P, based on Gradient Boosting and Support Vector Regression (SVR), were also tested during preliminary experiments. They were, however, less well-behaved on this dataset, and these results were omitted from further analysis due to their overfitting behaviors and low interpretability.

### 3.7. Evaluation Metrics

The dataset was split into training and test sets at an 80:20 split with randomization to evaluate the model performance. A sum of N=720 of samples was employed to train the model, and N=180 samples were left for evaluation. All models were trained on consistent splits for comparison.

Let $y_{i}$ and ${\hat{y}}_{i}$ be the true and predicted volumes for sample *i*, respectively, and suppose *n* to be the number of test samples. The true volumes mean is $\bar{y}=\frac{1}{n}\sum_{i=1}^{n}y_{i}$. The models ware assessed using the following metrics:

#### 3.7.1. Coefficient of Determination ($R^{2}$)

The coefficient of determination quantifies the amount of variance in the true volumes explained by the model:

(2) $$R^{2}=1-\frac{\sum_{i=1}^{n}{\left(y_{i}-{\hat{y}}_{i}\right)}^{2}}{\sum_{i=1}^{n}{\left(y_{i}-\bar{y}\right)}^{2}}.$$

#### 3.7.2. Mean Absolute Percentage Error (MAPE)

MAPE measures the average relative prediction error in percentage form:

(3) $$\mathrm{MAPE}=\frac{100\%}{n}\sum_{i=1}^{n}\left|\frac{y_{i}-{\hat{y}}_{i}}{y_{i}+\varepsilon }\right|,$$

where ε is a tiny constant (here $\varepsilon =10^{-6}$) included to avoid division by zero.

#### 3.7.3. Root Mean Square Error (RMSE)

RMSE provides an absolute prediction error in similar units to the target variable:

(4) $$\mathrm{RMSE}=\sqrt{\frac{1}{n}\sum_{i=1}^{n}{\left(y_{i}-{\hat{y}}_{i}\right)}^{2}}.$$

#### 3.7.4. Mean Bias

The mean bias (mean prediction error) mirrors systematic overestimation vs. underestimation:

(5) $$\mathrm{Bias}=\frac{1}{n}\sum_{i=1}^{n}\left({\hat{y}}_{i}-y_{i}\right).$$

Negative values are systematic underestimation, while positive ones reflect overestimation.

#### 3.7.5. Mean Intersection-over-Union (mIoU)

For segmentation performance, let $P_{c}$ and $G_{c}$ denote both the predicted and ground truth pixel sets for class c∈{1,…,C}. The Intersection-over-Union (IoU) for class *c* is

(6) $${\mathrm{IoU}}_{c}=\frac{|P_{c}\cap G_{c}|}{|P_{c}\cup G_{c}|},$$

and the mean IoU over the total of *C* classes is

(7) $$\mathrm{mIoU}=\frac{1}{C}\sum_{c=1}^{C}{\mathrm{IoU}}_{c}.$$

#### 3.7.6. F1 Score

For every class *c*, let ${\mathrm{TP}}_{c}$, ${\mathrm{FP}}_{c}$, and ${\mathrm{FN}}_{c}$ represent the true positives, false positives, and false negatives, respectively. Precision and recall are defined as

(8) $${\mathrm{Precision}}_{c}=\frac{{\mathrm{TP}}_{c}}{{\mathrm{TP}}_{c}+{\mathrm{FP}}_{c}},\;{\mathrm{Recall}}_{c}=\frac{{\mathrm{TP}}_{c}}{{\mathrm{TP}}_{c}+{\mathrm{FN}}_{c}},$$

and the F1 score for class *c* is

(9) $$F1_{c}=\frac{2\times {\mathrm{Precision}}_{c}\times {\mathrm{Recall}}_{c}}{{\mathrm{Precision}}_{c}+{\mathrm{Recall}}_{c}}.$$

We report per-class F1 scores, and we can take an overall summary by averaging across classes.

## 4. Results

The experimental evaluation was conducted on an augmented dataset of 900 mangosteen images, which were split into training (*N* = 720) and test (*N* = 180) sets. Four regression models were benchmarked using features extracted from multi-view instance segmentation.

### 4.1. Segmentation Performance and Feature Extraction

**Figure 6:**
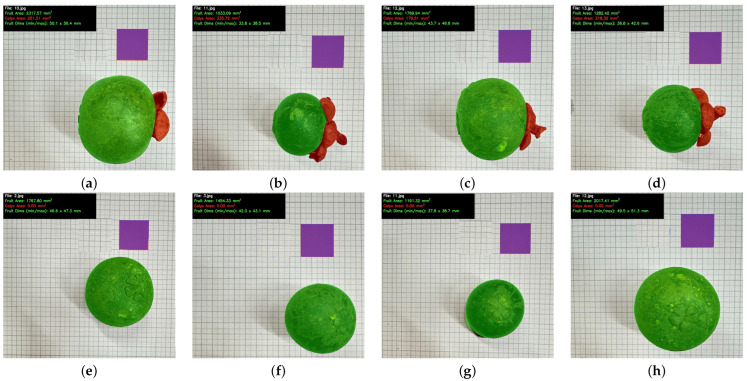
Example outputs of the instance segmentation model. Top row (**a**–**d**): Side-view images showing the clear separation of the fruit body (green) from the calyx (red). Bottom row (**e**–**h**): Bottom-view images showing the segmentation of the fruit body and marker (purple).

The YOLO-based instance segmentation model was successfully applied to both side- and bottom-view images and produced consistent detection and pixel-accurate masks for the three target classes: mangosteen (fruit body), calyx, and marker. Qualitative examples from Figure 6 show the model reliably separated the quasi-spherical fruit body from the irregular calyx, which we identified as a primary source of geometric noise for subsequent volume estimation tasks.

Segmentation quality was measured by mean Intersection-over-Union (mIoU) and per-class F1 score on a held-out segmentation validation set. The model obtained mIoU = 0.91, and the F1 scores were 0.94 (mangosteen), 0.88 (calyx), and 0.97 (marker) for each class. The lower performance for the calyx class reflects its morphological variability and partial occlusion in some side views. The mangosteen masks had to be isolated precisely from the calyx mask, since inclusion of calyx pixels in area/dimension calculations markedly degraded regression model performance (as illustrated below).

Features were extracted from the validated, cleaned, and augmented dataset (total N=900 images). After random stratified splitting of the available data into training (N=720) and test (N=180) sets, the predictive model evaluation discussed here relies only on the unseen test set.

### 4.2. Comparison of Regression Models

We then built and tested eight regression methods, which were categorized into single-view geometric model, multi-view geometric combination, ellipsoid approximation, hybrid linear combination, and sophisticated machine learning regressors (Table 4). Testing set performance variables (N=180) were coefficient of determination ($R^{2}$) and MAPE. Finally, we also reported RMSE and mean bias (mean prediction error) in our calculations for completeness. Key observations are as follows:

- The bottom-only model (G) slightly outperformed the side-only model (B); this means that the bottom projection encodes marginally better volume cues for mangosteen orientation utilized in this dataset.
- All of the multi-view models (I, L, N) significantly outperformed respective single-view ones, confirming the contention that orthogonal projections encode complementary shape information.
- M approach had a poorer accuracy than several data-driven methods ($R^{2}=0.6695$, MAPE = 19.62%) despite being based on two dimensions derived from views. However, a rigid ellipsoid assumption does not account for the constant deviations in the cross-sectional form of mangosteen fruits from ellipses, particularly for those bearing prominent calyx lobes and asymmetric shoulders.
- The hybrid linear model (N), which linearly fuses both area-based features ($A_{s}^{1.5},A_{b}^{1.5}$) and the ellipsoid feature ($V_{ellipsoid}$), achieved the best accuracy/correlation ratio for accuracy and bias-correction (highest $R^{2}=0.7241$, lowest MAPE = 16.72%, and least RMSE and bias).
- Advanced ML models (Gradient Boosting O, SVR P) also did not outperform simple combined linear ones. We take this as a signal that volume of data is strongly geometric and very low-dimensional; higher-capacity models can overfit to the training distribution or need a greater variety of examples for generalization.

**Figure 7:**
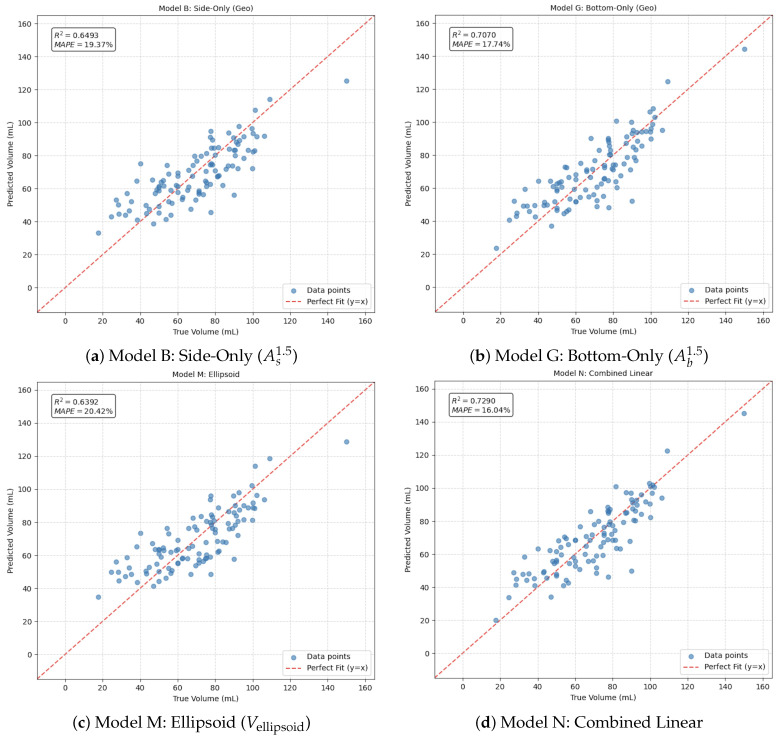
(**a**–**d**) show the Predicted versus true volume on the test set for selected regression models (B, G, M, N). Each scatter plot compares predicted and ground truth volumes. The red dashed line represents the ideal prediction line (y=x). Model N (**d**) demonstrates the tightest clustering around the identity line, indicating superior predictive performance.

Scatter plots of predicted vs. true volume of representative models are shown in Figure 7. Model N exhibits the narrowest clustering about that identity line and the smallest systematic deviation.

### 4.3. Residual Analysis and Diagnostic Tests

**Figure 8:**
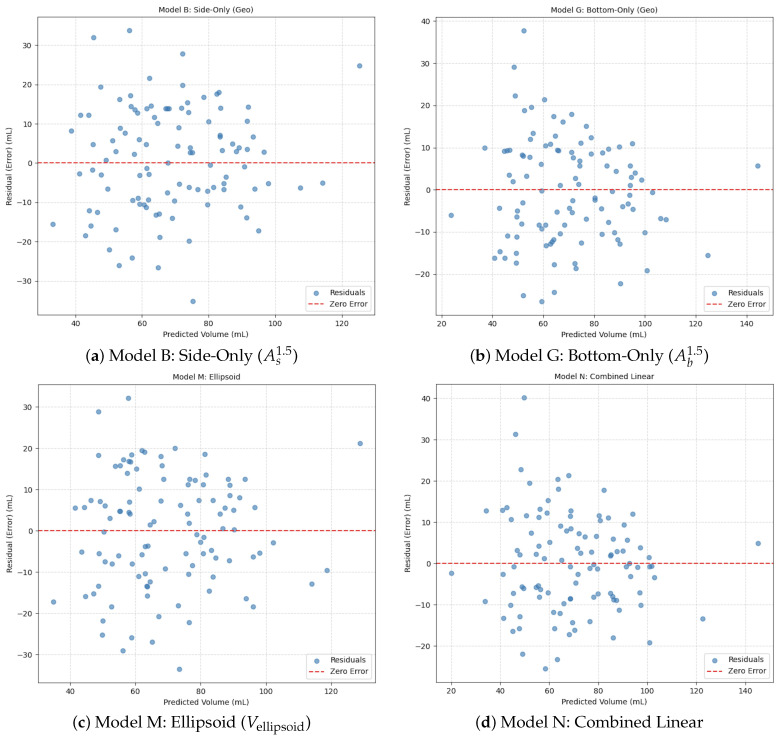
(**a**–**d**) show the Residual plots comparing predicted volume versus residual error for selected regression models (B, G, M, N). The horizontal red dashed line at zero represents perfect prediction (no error). Model N (**d**) demonstrates the most desirable residual distribution—randomly scattered around zero with minimal heteroscedasticity—indicating superior generalization and model fit.

We carried out residual analysis following the error definition $e=V_{\mathrm{true}}-V_{\mathrm{pred}}$, where $V_{\mathrm{true}}$ is ground truth fruit volume and $V_{\mathrm{pred}}$ is model-predicted volume, to test the robustness of the model and identification of systematic errors. Simultaneously, unbiased, inconsistent, and agreeant statistical tests between models were included in the analysis. To the best of our knowledge, a visual inspection of residual plots (Figure 8) revealed some trends regarding the different modeling approaches:

- **Model N** (hybrid multi-view regression) produced residuals with relatively homoscedastic properties and symmetric distribution about 0, reflecting that there was no size-dependent bias and stable estimates were obtained over the fruit size interval.
- **Model M** (ellipsoid-based regression) displayed significant heteroscedasticity with respect to residual variance scaling with predicted volume. The ellipsoid approximation does not scale well for larger fruits, and it can over- or under-estimate volume based on shape deviations.
- **Single-view models** resulted in higher residual distributions with less predictability and dependency on perspective.

To verify these observations, we conducted a series of statistical tests to assess predictive accuracy, variance stability, and agreement with ground truth.

- **Paired*****t*****-test** for the absolute errors of Model N and Model I (only side view) was p<0.01, showing that predictive accuracy improvements reported by Model N are statistically relevant at a 1% level.
- **Levene’s test** for the equality of variances between residuals from Model N and Model M led to p<0.001, lending more confidence in heteroscedasticity in the ellipsoid model, and justifying the larger variance stability found in Model N.
- **Bland–Altman analysis** shows that Model N induced a mean bias close to zero and narrower 95% limits of agreement than Model M and single-view models. This means more agreement with ground truth over all of the dynamic range of fruit size.

Collectively, these diagnostic results strengthen the hybrid multi-view method and demonstrate the limitations of geometric approximations in irregular fruit morphologies in tropical countries.

### 4.4. Residual and Distributional Diagnostics

**Figure 9:**
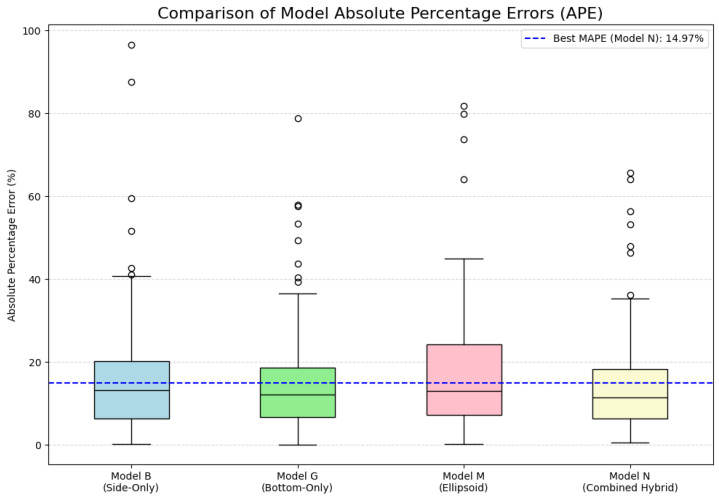
Boxplot comparison of absolute percent errors for four regression models. Model N (Combined Hybrid) shows the narrowest interquartile range and lowest median error, indicating superior robustness. Model M (Ellipsoid) exhibits the widest spread and highest upper quartile, reflecting its sensitivity to shape deviations. Models G and B (Bottom-Only and Side-Only, respectively) confirm that bottom-view features yield more reliable predictions than side-view alone.

We tested the robustness of the regression models by studying the absolute percent errors across four representative configurations. Absolute percent error graphs were plotted in Figure 9 and detailed statistics such as mean, standard deviation (SD), quartiles, as well as extremes were given in Table 5. This review will strengthen the residual and statistical analysis and give extra context on maintaining consistent performance beyond how the system performs on average.

Model N (Combined Hybrid) presented the best accuracy and stability, but had low mean error (14.97%), had a 11.44% error median range and the smallest interquartile range (Q1–Q3: 6.40–18.25%), as well as the lowest observed maximum error (65.66%). In contrast, Model M (Ellipsoid) showed the largest error difference and highest upper quartile (Q3 = 24.22%), which can be associated with its sensitivity to morphological deviance and its limited scalability for larger fruit specimens. Models G and B (Bottom-Only and Side-Only, respectively) performed slightly better, and had achieved the optimal performance when using Model G, still outperforming Model B only, and these results further confirm earlier work showing that bottom-view features result in higher predictability of fruit quantity than on side-view features. Taken together, these distributional diagnostics demonstrate that, for mangosteen volume estimation, hybrid features are the most balanced and credible over both standard geometric approximations or single-view regressors.

### 4.5. Difficult Cases and Analysis of Failure Cases

**Figure 10:**
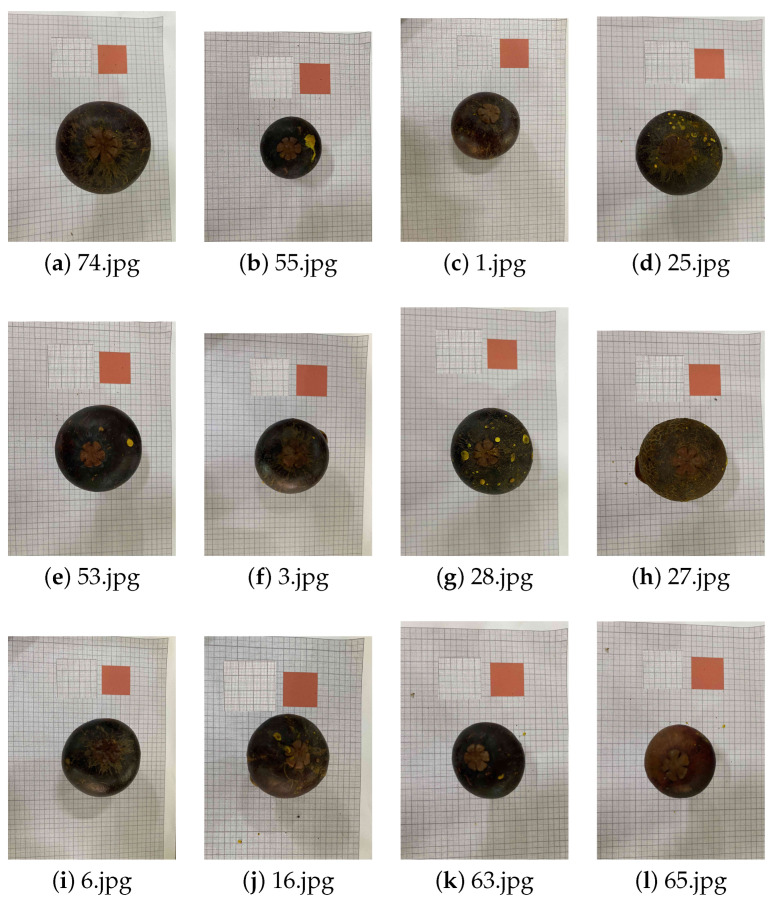
Challenging bottom-view examples from the dataset. Images (**a**–**l**) correspond to extreme-volume fruits that lie far from the dataset mean, including very large cases such as (**a**) 74.jpg (150 mL) and very small cases such as (**b**) 55.jpg (30 mL). These extremes amplify the impact of segmentation imprecision and scale-calibration noise, making them representative stress-test cases for evaluating robustness of the proposed workflow.

To evaluate robustness under non-ideal conditions, we analyzed the bottom-view samples showing the largest deviations from the mean ground truth volume. Representative challenging cases are displayed in Figure 10, panels (a)–(l). These fruits lie at the upper and lower extremes of the volume distribution, including very large samples such as panel (a) (150 mL) and very small samples such as panel (b) (30 mL). Such extremes magnify the effect of boundary noise and scale calibration uncertainty and are informative stress-test scenarios for the proposed workflow. In spite of these variations, the YOLO-based instance segmentation model generally produced clean fruit body masks, even when the bottom-view projection was highly compressed or nearly filled the frame.

## 5. Discussion

The experimental results provide a number of methodological and practical guidelines for computerized fruit phenotyping, especially for composite fruits like mangosteen, with erratic morphology and conspicuous calyx structures.

### 5.1. Efficient Isolation of Fruit Components Is Essential

Initial experiments that did not separate the calyx from the fruit body mask resulted in markedly reduced regression performance ($R^{2}<0.40$). The calyx produces morphological noise at the area- and contour-related descriptors, showing once again that exact instance segmentation is a prerequisite for reliable volume estimation. An explicit object class-modeling of a calyx ensures that irregular geometry is not further generalized to quantitatively representative fruit.

### 5.2. Multi-View Attributes Enhance Performance but Will Need Meaningful Integration

As a single-view descriptor only gives meaningful glimpses into the shape of the fruit, orthogonal perspectives (side and bottom views) produce a much clearer predictive mechanism. But a simple additive or polynomial combination of $A_{s}^{1.5}$ and $A_{b}^{1.5}$ were inadequate to fully explain variability in fruit geometry. The best accuracy was obtained when combinations of these description details were applied to a parametric geometric prior such as the ellipsoid-based volume estimate ($V_{\mathrm{ellipsoid}}$) in a regularized linear regression model (Model N), illustrating the importance of jointly modeling multiple views and structured shape priors.

### 5.3. Limitations of Ellipsoid Approximations for Irregular Morphology

Ellipsoid-based formulations have been proved effective with spherical fruits, e.g., oranges [48], and have proven effective with hand-measured mangosteen datasets [20,21]. On the other hand, using automated image-derived measurements across a larger and more variable sample set, the same formula (Model M) showed less accuracy ($R^{2}=0.6392$, MAPE = 20.42%). The discrepancy is due to larger sample variability, lower measurement accuracy than caliper-based procedures, and shape deviations due to calyx. It suggests that while $V_{\mathrm{ellipsoid}}$ still serves as a useful descriptor, we require multi-view geometric features ($A_{s}^{1.5}$, $A_{b}^{1.5}$) to make robust predictions. The hybrid model (Model N) reached $R^{2}=0.7290$ and MAPE = 16.04%, illustrating the cost-effectiveness of combining heterogeneous geometric cues.

### 5.4. Interpreters Can Surpass Complex Regressors

Even when there are more advanced machine learning models like gradient boosting and support vector regression, we found out that the standard linear regression model (Model N) performed the best and has been the most stable. This highlights the need of domain-informed feature engineering and easily interpretable modeling methods, especially in context of scarce datasets or infrastructure deployment issues that are for transparency purposes and computational need for high-speed.

### 5.5. Background, Occlusion, and Scale Issues

While the experiments were performed in a laboratory-like setup, real conditions add other limitations that must be considered. Blurred or disorderly backgrounds can lower segmentation performance due to the creation of contour lines, texture details similar to fruit boundaries. Partial occlusion (e.g., calyx lobes that cover parts of the fruit) or inter-fruit obstruction (e.g., near-fruit presence in the processing lines) can affect the extracted features and lead to poor regression performance. In addition, scaling deviation due to varying camera-to-object distance can lead to geometrical bias unless reliable per-image calibration markers or depth cues are provided. Dealing with these factors might warrant stronger augmentation techniques, background-invariant segmentation models, or an adaptive scale normalisation method to make it more robust, independent of controlled imaging environments.

### 5.6. Operational Accuracy and Practical Convenience

While the best-performing model did not yield the high accuracy attained for geometrically normal fruits ($R^{2}\approx 0.94$ for oranges for example), it produced an acceptable ($R^{2}=0.7241$, MAPE = 16.72%) in coarse size grading (small–medium–large) and provides potential benefits to post-harvest sorting operations. A MAPE between 15 and 18% is acceptable for operations and is a marked upgrade from manual methods, notably for those fruits with irregular morphology.

**Overall importance.** This work shows that accurate (interpretable) volume estimation for mangosteen can be performed using inexpensive 2D imaging, multi-view instance segmentation techniques, and hybrid geometric modeling. The proposed framework eliminates 3D reconstruction, depth sensing, and manual measurement by using ellipsoid-derived descriptors and projected-area features. The constructed framework is efficient for computation scale-up to high-throughput environments and for integration into automated grading and quality-control pipelines for tropical fruit plantations.

Although the proposed framework demonstrates strong potential for practical deployment, several limitations remain that highlight opportunities for future research:

- **Not many views.** Side and bottom views are used to give complementary information, but they may not be able to cover full dimensions of shape complexity. The availability of more oblique views will add value but can supplement it and reduce the overall volumetric fidelity, augmenting the system with cheap depth-sensing technologies.
- **Occlusion and variances of calyx.** Discrepancy shape of calyx and partial occlusion on side views continue to be the challenges for segmentation. Attention-based or transformer-based segmentation architectures could be used to enhance robustness with occlusion in future work.
- **The diversity of the dataset.** The dataset does not currently cover the full range of cultivars, maturity stages, and environmental settings, although further data augmentation has increased the variability. More diverse and larger datasets would result in better generalization.
- **Modeling hybrid strategies.** If geometric priors and relatively lightweight neural networks can also be paired to learn residual corrections, accuracy could be enhanced without compromising interpretability and effectiveness.

## 6. Conclusions

A new non-destructive volume estimation framework based on multi-view instance segmentation and hybrid geometric feature modeling for mangosteen fruit was proposed in this novel approach and was validated and tested for mangosteen fruit in this work. The method integrates deep learning-based image analysis and interpretable regression methods, facilitating future prospects for automated fruit size automation in agricultural processing pipelines.

A major outcome of this study is the inclusion of instance segmentation to more clearly divide the fruit body from the calyx, improving the composite fruit body feature extraction performance. Single-view models are limited but multi-view models using orthogonal projections did better than single-view approaches. Moreover, for mangosteen, due to morphological irregularity and calyx variability, classical ellipsoid approximations (which are also applicable for regular-shaped fruit) were not adequate.

The best prediction is obtained by a hybrid linear regression model (Model N) that combines the side-view and bottom-view area features ($A_{s}^{1.5}$ and $A_{b}^{1.5}$) with an ellipsoid-derived volume estimate ($V_{\mathrm{ellipsoid}}$), with a predictive result of $R^{2}=0.7290$ and MAPE=16.04% for tested data. These results indicate that coupling complementary geometric descriptions with domain-based priors promotes robustness and model interpretability, allowing the proposed framework to be implemented in real time in sorting and grading schemes.

Acknowledging the power of the framework, it does have important limitations that could be explored further. First, since irregular fruits are three-dimensional, more perspectives or lightweight depth sensing methods could enhance volumetric fidelity. Second, segmentation accuracy still depends on calyx occlusions and morphology, which indicates the need for more sophisticated architectures (attention/transformer-based segmentation models, etc.) that could provide better reliability. Third, while augmented, we have only a limited representation of cultivars, maturity stages, and environmental variables. Therefore, a larger and more heterogeneous dataset will reinforce generalization. Addressing these issues through improved imaging, more stable segmentation models, and larger datasets will allow the framework to be adapted for wider operational scenarios and to address different fruit types.

This work is ultimately a scalable and interpretable yet low-cost implementation using standard 2D imaging for fruit volume estimation; it has practical relevance for assessing post-harvest quality and aims to pave the way forward for future implementations of solutions in smart agriculture systems.

## Figures and Tables

**Table 1:** Descriptive statistics for the cleaned validation dataset (N=419).

Statistic	Mass (g)	Volume (cm**^3^**)
Mean	55.37	51.52
Std. Dev.	10.88	10.61
Min	35.80	30.00
25% (Q1)	47.60	43.00
50% (Median)	54.90	52.00
75% (Q3)	62.80	59.00
Max	86.50	78.00

**Table 2:** Summary of image preprocessing and augmentation parameters applied during YOLOv8 model training.

Preprocessing Step	Details
Auto-Orientation	Applied to correct image rotation metadata
Resize	Stretched to 640 × 640 pixels
Contrast Adjustment	Histogram Equalization
Augmentation Type	Parameter Range/Description
Outputs per Image	3 augmented variants per training example
Flip	Horizontal
Rotation	90° clockwise and counter-clockwise
Hue Shift	Between $-15^{{}^{\circ}}$ and $+15^{{}^{\circ}}$
Saturation Variation	Between −25% and +25%
Brightness Adjustment	Between −15% and +15%
Exposure Shift	Between −10% and +10%
Gaussian Blur	Up to 2.5 pixels
Salt-and-Pepper Noise	Up to 0.1% of pixels

**Table 3:** Summary of extracted geometric features derived from segmented fruit masks.

Feature	Definition	Unit
$A_{s}$	Side-view fruit area	cm^2^
$A_{b}$	Bottom-view fruit area	cm^2^
$A_{s}^{1.5}$	Power-law transformation of $A_{s}$	cm^3^
$A_{b}^{1.5}$	Power-law transformation of $A_{b}$	cm^3^
*h*	Fruit height (side view)	cm
*w*	Fruit width (bottom view)	cm
$d_{1}$	Depth estimate from side view	cm
$d_{2}$	Depth estimate from bottom view	cm
$V_{e}$	Ellipsoid volume estimate	cm^3^

**Table 4:** Performance comparison of all regression models on the unseen test set (N=180). Best performance for each metric is in **bold**.

Category	Model	$R^{2}$	MAPE (%)	RMSE (cm^**3**^)	Bias (cm^**3**^)
Single-View	B: Side-Only ($A_{s}^{1.5}$)	0.6493	19.37	36.4	−2.5
Single-View	G: Bottom-Only ($A_{b}^{1.5}$)	0.7070	17.74	34.1	−1.9
Multi-View	I: Geo combined ($A_{s}^{1.5}+A_{b}^{1.5}$)	0.7121	17.68	33.5	−1.6
Multi-View	L: Poly-Geo (deg = 2)	0.7114	17.68	33.7	−1.8
Ellipsoid	M: $V_{ellipsoid}$	0.6392	20.42	37.8	−4.2
Hybrid	**N: Combined Linear (areas + $V_{\mathrm{ellipsoid}}$)**	**0.7290**	**16.04**	**31.9**	**−0.8**
Advanced ML	O: Gradient Boosting	0.6679	19.02	35.6	−2.1
Advanced ML	P: SVR (RBF)	0.5340	20.85	42.9	−5.8

**Table 5:** Boxplot statistics of absolute percentage error on the test set (N=180).

Model	Mean	Std. Dev.	Min	Q1	Median	Q3	Max
B: Side-Only	16.51	15.89	0.16	6.37	13.26	20.20	96.60
G: Bottom-Only	15.46	13.65	0.09	6.63	12.22	18.71	78.83
M: Ellipsoid	16.95	15.54	0.18	7.29	13.08	24.22	81.78
N: Combined Hybrid	14.97	13.54	0.61	6.40	11.44	18.25	65.66

## Data Availability

The raw data supporting the conclusions of this article will be made available by the authors on request.
